# Defining Content for a Competency-based (CanMEDS) Postgraduate Curriculum in Ambulatory Care: a Delphi Study

**Published:** 2012-03-31

**Authors:** René Wong

**Affiliations:** University of Toronto, Toronto, Ontario, Canada

## Abstract

**Background:**

Ambulatory training in internal medicine has been noted to be dysfunctional and inadequate. In this study, we developed a set of competency-based outcomes specific to ambulatory care to guide the design, implementation and evaluation of instructional events to ensure that societal needs are addressed.

**Methods:**

In 2007 a Delphi technique was used to reach consensus and define the priorities for competency-based training in ambulatory care for internal medicine residents. Four groups of stakeholders in Canada participated: program directors, members of the Canadian Society of Internal Medicine, recent graduates, and residents.

**Results:**

Two rounds of the Delphi process were required to reach consensus on a set of sixty competency-based educational objectives in ambulatory care that were classified under the CanMEDS roles. The inclusion of recent graduates in this study resulted in the addition of non-clinical topics that would have otherwise been missed, falling under roles historically viewed as being challenging to teach and evaluate (Manager, Health Advocate).

**Conclusion:**

This study is the first time a Delphi-process has been used to define the priorities for ambulatory care training in internal medicine under a competency-based framework. The resulting compendium of competency-based objectives provides a foundation from which educators can design, evaluate and modify existing training experiences.

## Introduction

Postgraduate medical education (PGME) programs must ensure that their graduates master a series of core competencies to better respond to societal needs. The core competencies of both the Accreditation Council for Graduate Medical Education (ACGME) in the United States and the Royal College of Physicians and Surgeons of Canada (RCPSC) have adopted frameworks describing the spectrum of professional competencies from which to set educational standards in program accreditation, resident assessment, and maintenance of competency.^1^,^2^ Ambulatory care has become a critical aspect of the health-care system, but ambulatory training in internal medicine (IM) PGME programs has been noted to be “dysfunctional” and “inadequate” by practicing physicians and professional organizations.^3^–^8^ Although it is stipulated that one-third of the training experience should be in the ambulatory setting, the majority of trainees’ time continues to be spent on inpatient wards.^2^ When comparing inpatient to ambulatory experiences, there are not only unique differences in the scope and acuity of clinical problems, but also of non-clinical topics for which calls have been made to improve the quantity and quality of teaching.^3^,^5^,^7^,^9^,^10^,^11^ It should not be assumed that learning experiences gained by working on inpatient wards can be generalized to the knowledge, skills and attitudes required for working in the ambulatory setting.^12^ The adoption of a competency-based framework for postgraduate ambulatory care training that reflects key clinical and non-clinical topics would help teach future specialist physicians about the multi-facetted roles expected of them. Programs could then appraise the ‘distance’ between high-priority goals and their current status when evaluating and modifying their ambulatory training programs.

The Delphi technique is a qualitative research method that is one of the most common and successful methods for identifying professional competencies.^13^ Consensus of opinion among a group of experts is attained without face-to-face discussion using a series of questionnaires administered by mail or electronically, with controlled feedback from the researchers after each round of questions.^14^ It avoids the pressures, biases and costs of face-to-face discussion and permits the use of more experts than would otherwise be possible, in particular when they are separated geographically. With successive iterations, responses tend to converge and eventually lead to a consensus.

In this study, a modified Delphi technique was used to establish training priorities for a competency-based curriculum in ambulatory care for IM residencies. Broad representation from a heterogeneous group of key stakeholders provided a wide range of perspectives.

## Method

Ethics approval was obtained from the local institutional research ethics board.

Between November 2006 and April 2007, panellists were invited to participate with the goal of generating a compendium of competency-based objectives in ambulatory care. The process was predetermined to continue until consensus was reached for every item.

### Round 1

In a traditional Delphi technique, experts are gathered to discuss and identify themes for potential competencies. This approach was revised by providing pre-existing information for ranking because it would have been logistically difficult to gather experts representing each stakeholder group to meet.^15^,^16^ An initial list of competencies was adapted from the objectives of training for core IM^17^ and a Medline search of the literature was conducted using the terms ‘postgraduate medical education’, ‘curriculum’ and ‘ambulatory care’.^3^,^12^,^18^,^19^ To enable integration into existing curricula, topics were categorized under the seven CanMEDS roles: Medical Expert, Communicator, Collaborator, Manager, Health Advocate, Scholar, and Professional. An initial list of 73 educational objectives was generated and included in the questionnaire for round one. Specific disease content was not included to keep competencies applicable to all IM subspecialties.

Broad representation of opinions was solicited from groups of stakeholders who may have differing perspectives on ambulatory education: 1) program directors and members of the IM specialty committee of the RCPSC, 2) members of the Canadian Society of Internal Medicine (CSIM), 3) recent graduates (within the prior five years) of IM training programs in clinical practice, and 4) residents in core IM residency programs in Canada. Open invitations were sent via email with a goal of recruiting a minimum of seven participants from each stakeholder group.^14^ There were no explicit exclusion criteria, preserving the integrity of the Delphi process.^20^ Reminders were sent to non-responders after four and, if necessary, again after eight weeks following the initial distribution of the first round. Participants were asked to recommend names of individuals who might be interested in participating.

Participants were asked to rate each objective based on the need to include it in an ambulatory-care specific curriculum during residency, using a five-point Likert scale (1 = Not important, can safely be omitted, 2 = Less important, probably exclude, 3 = Uncertain, 4 = Important, probably include, 5 = Essential for ambulatory care program, definitely include). The criteria for inclusion, exclusion, and reaching consensus were adapted from published reports.^21^–^23^ Consensus for each item was predefined to be reached if the difference between the 25^th^ and 75^th^ percentile values of the panel’s ratings was equal to or < 1. Any item that met consensus with a median score of 5 and a minimum of 75% agreement amongst the respondents (i.e. > 75% rated it as 4 or 5) was included in the final compendium as ‘Priority 1 (Must be able to)’. Any item that met consensus with median scores of 1 or 2 with a minimum of 75% agreement was excluded. All other ratings (i.e. those not fulfilling the criterion for consensus), including items for which consensus was met but with median scores of 3 or 4, were included into the next round. Panellists were invited to make suggestions for topics not already included, clarify content, and identify objectives that seemed irrelevant to the project.

### Round 2

Panellists were asked to rate the remaining items using the same five-point scale used in the previous round. The respective median and interquartile limits (from round 1) were shown for each item. Items for which consensus was met, with median ratings of 1, 2 or 3, were excluded. Items that met consensus with median ratings of 4 or 5 were included in the final compendium and assigned priority to ascertain the strength of these ratings relative to one another.^21^

*Priority 1 (Must be able to)*: Median of 5, with a mode of 5 rated by over 75% of respondents.
*Priority 2* (*Should be able to)*: Median of 4, with > 75% of respondents rating it 4 or 5.
*Priority 3 (Would be nice if able to)*: Median of 4, with 50–75% of respondents rating it 4 or 5.

Remaining items for which consensus was not reached were kept to be included in subsequent rounds and questionnaires, using the same criteria for inclusion (and priority classification), exclusion, and subjection to further rounds.

### Data analysis

Spearman rank order correlation coefficients were generated on the rank orders between the groups. ‘Fountain graphs’ that simultaneously plot the standard deviations against the means for all items were created as a way to illustrate both the overall distribution of opinions and the extent of agreement at each round.^24^

## Results

After initial email recruitment, 424 physicians agreed to participate. A total of 73.6% of the practicing physicians in the panel were involved in teaching ambulatory care. Demographic data of the panel are shown in Table 1.

After round one, 19 competencies met the inclusion criteria as priority level one topics and nine met the criteria for exclusion. Two new competencies were added, and two were noted by multiple panellists as too similar to others and were consequently deleted. The remaining 45 items were subsequently included in round two.

Thirty-six participants from round 1 indicated that they did not wish to complete further questionnaires. Of the remaining 388 participants, 240 (61.86%) completed the round two questionnaire. Using the pre-defined criteria, consensus was reached for all remaining items and consequently no further rounds were required.

### Competencies identified for the framework

Table 2 shows the 21 topics classified as priority level one for inclusion in the compendium, most under the roles of Medical Expert, Communicator, and Professional. A further 27 topics and 12 topics were classified as priority levels two and three, respectively (Tables 3 and 4) for inclusion. Select competencies under the Manager and Health Advocate roles (indicated in Tables 3 and 4) would have been missed if the recent graduates had not been included in the study.

### Comparisons between groups

Spearman rank order correlation correlations among the rankings of the four groups ranged from 0.90 to 0.95, indicating similar assessment of the importance of items among the respondents.

### Stability of responses

**Figure 1:**
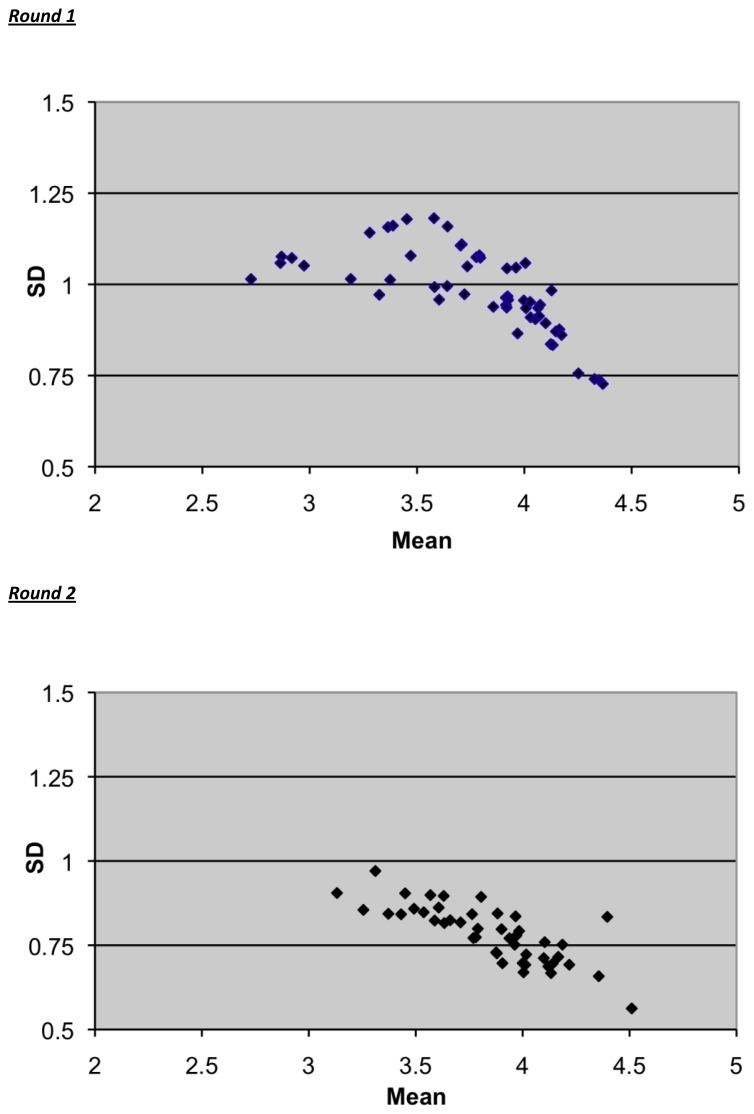
Fountain graphs displaying means and standard deviations (SD) for all items in Rounds 1 and 2.

Figure 1 shows ‘fountain graphs’ for rounds one and two. There was a more sharply focused pattern for round 2, with lower standard deviations indicating stabilization of opinion and high consensus among the group. Greater changes in distributions would have represented low consensus among the group and a need for subsequent rounds to increase the agreement within the group.

## Discussion

This study reports the results of a comprehensive Delphi process used to reach consensus on clinical and non-clinical training needs in ambulatory care using a competency-based framework. Even though the study was conducted in Canada, the CanMEDS roles have been adopted and adapted by many jurisdictions and health professionals worldwide. It can be applied to the U.S. system because of the similarities between the CanMEDS and ACGME frameworks. Barker^12^ and Robbins^19^ previously developed guidelines to design and implement curricula in ambulatory care for IM residents in the U.S. As both were published prior to the development of the ACGME core competencies, however, the authors would not have been able to classify them accordingly. More recently, a taskforce operationalized the six ACGME competencies with specific behavioural milestones using a developmental framework.^20^ The majority of the identified Level 1 competencies in this study are similar to those included in that framework and are not unique to ambulatory care. The results of this study differ by providing a more focused definition of non-clinical competencies, notably under the Manager and Health Advocate roles, that are not as explicitly described in the ACGME framework.

A broad inclusion of a heterogeneous group of key stakeholders guarantees a wide range of knowledge and perspectives.^20^,^25^ A unique aspect of this study is the enhanced breadth of information obtained by the inclusion of recent graduates who are frequently not included in curriculum planning. Having recently completed residency training and started their clinical practices, they can identify topics for which they have been inadequately trained that may not be otherwise considered by traditional curriculum planners. This was exemplified in this study; the identification and definition of select competencies under the CanMEDS roles of Health Advocate and Manager (Tables 3 and 4) would not have occurred if recent graduates had not been included in the Delphi process. These roles have been historically viewed as confusing and challenging to teach and evaluate in general, let alone specific to the ambulatory setting.^26^,^27^,^28^ By highlighting specific competencies under these roles, it is now possible to define measurable behaviours that could subsequently be used to determine competency relevant to the ambulatory setting.

The rigor of this study is supported by using a predetermined decision trail for the inclusion and exclusion of topics based on published work,^21^,^23^ but allowing a degree of openness to the responses by encouraging panellists to make suggestions to the preliminary list. It was somewhat surprising that in this study only two rounds were required to reach consensus, although this could likely be attributed to an initial compilation of topics that was already well-focused through a large degree of review. The stability of the distributions of responses over successive rounds shown in this study strengthens the richness of information over and beyond reaching consensus.^25^,^29^ The fountain graphs show lower standard deviations and a narrower range of ratings with subsequent rounds demonstrating a state of equilibrium. Greater changes in distributions would have represented low consensus among the group and a need for subsequent rounds to increase the agreement within the group.

There is a wide variation in numbers of participants in published Delphi studies with reports ranging from 10 to over 1000.^30^ The intent of this study was to select a convenience sample of a minimum number from each key stakeholder group and not exclude participants from any stakeholder group, in order to ensure a wide range of perspectives. While the number of participants in this study is greater than for typical Delphi studies, similar sample sizes have been reported.^31^,^32^ A larger sample size, moreover, increases the reliability of the method.^33^ The demographics of the panel with respect to sex and specialty were similar to that of internists certified by the Royal College of Physicians and Surgeons of Canada,^34^ suggesting good generalizability of the findings. The characteristics of the round two responders compared to the non-responders with respect to sex, years in practice, subspecialty (general medicine or subspecialty) were similar, and the low attrition rate was reassuring. The remarkable consensus achieved in round two provides validity of the conclusions drawn from the data.

This study has limitations. As in any cross-sectional study, the opinions expressed are those for single points in time. It is also possible that responses may have been influenced by the way in which the topics were written or by omission of topics in the preliminary list. However, all panellists were invited to make suggestions for topics that were not included in the first iteration. Lastly, the Delphi process achieves consensus and may minimize the impact of opinions held by a minority.

### Conclusion

This is the first time that a Delphi method, using opinions of key stakeholder groups including recent graduates, was used to reach a consensus on competency-based training needs for IM residents for ambulatory care. It identified and defined previously underemphasized non-clinical topics, notably under the CanMEDS Manager and Health Advocate roles that have been traditionally difficult to teach and evaluate. It advances the work on competency-based graduate medical education in the outpatient setting and provides a foundation from which educational planners can develop behavioural milestones to measure and evaluate residents’ performance. Further research to develop and validate tools to teach and evaluate these topics is needed.

## Figures and Tables

**Table 1:** Characteristics of participants.

Characteristic	%	Number
**Panellists (n = 424)**		

- Program directors and members of the Royal College Specialty Committee for Internal Medicine	8.7	37
- Canadian Society of Internal Medicine members	25.5	108
- Recent graduates	16.7	71
- Current residents	49.1	208
- Sex		
- Female	41.5	176
- Male	51.4	218
- Not specified	7.1	30

**Specialty (practicing physicians only)**		

- General internal medicine	56.0	121
- Medical subspecialty	38.8	84
- Not specified	5.2	11

**Teaching role in the ambulatory setting (practicing physicians only)**		

- Supervises residents/students	73.6	159
- Does not supervise residents/students	15.7	34
- Not specified	10.7	23

**Practice type (practicing physicians only)**		

- Affiliated with a hospital	57.4	124
- Not affiliated with a hospital	28.2	61
- Not specified	14.4	31

**Duration of practice (practicing physicians only)**		

- 5 years or less	43.0	93
- Greater than 5 years	46.8	101
- Not specified	10.2	22

**Table 2:** Competencies classified as level one priorities

**Medical Expert**
- Generate an appropriate differential diagnosis and management plan a - Appropriately select diagnostic tests with an understanding of their utility, limitations and complications a - Conduct an accurate and focused history and physical examination a - Describe risks and benefits of treatment options when discussing a management plan with a patient a - Demonstrate medical expertise when providing ongoing care to a patient with a chronic problem that is unstable a - Demonstrate medical expertise when managing a patient in preparation for surgery a - Appropriately prioritize requests for outpatient consultation from other health care providers based on urgency
**Communicator**
- Create an effective consultation letter to the referring physician in an efficient manner a - Interact with patients in a manner that respects their concerns, expectations and confidentiality a - Maintain clear, accurate and appropriate records (written or electronic) of clinical encounters and plans a - Present information to patients in a way that encourages discussion and autonomy a - Appropriately respond to anger, confusion or misunderstanding from a patient, family member or other health care provider a - Effectively present verbal reports of clinical encounters and/or management plans to another health care provider a
**Collaborator**
- Upon discharge of a patient from his/her practice, create a plan for ongoing management in collaboration with the primary care physician a
**Manager**
- Effectively balance time between professional and personal/home life a
**Professional**
- Treat patients and their families with compassion and respect regardless of sex, ethnicity and/or cultural issues a - Recognize and accept limitations in knowledge/ability and refer patients for a second opinion when appropriate a - Treat other physicians and health care providers in a collegial and respectful manner a - Determine when and how to end a physician/patient relationship a - Describe the legal, ethical and professional requirements for the disclosure of medical errors a - Describe the principles and limits of patient confidentiality a

a indicates competencies that met inclusion criteria after one round

**Table 3:** Competencies classified as level two priorities

**Medical Expert**
- Demonstrate medical expertise when following a patient longitudinally over multiple visits - Describe properties of commonly-used drugs, including mechanisms of action, adverse effects and potential drug interactions - Select appropriate time intervals for follow-up appointments - Demonstrate medical expertise when providing medical advice to patients or other health care professionals over the telephone - Demonstrate medical expertise when providing advice to a patient regarding his/her fitness for work, driving and/or exercise - Perform procedures in the office/clinic setting in an effective and timely manner - Demonstrate medical expertise when providing end-of-life care to a patient
**Communicator**
- Accurately document discussions with patients that occurred via telephone - Provide useful feedback to office/clinic staff and/or students/residents
**Collaborator**
- Consider, accept and respect the opinions of other multidisciplinary team members while discussing medical or social issues of a patient - Work with other health care professionals to prevent and resolve conflict
**Manager**
- Effectively balance time between professional activities (e.g. patient care, paperwork, teaching, administration, research etc.) - Principles of physician remuneration, including the billing process, third-party billing and billing for uninsured services - Design an effective appointment system that assures timely appointments and an appropriate volume of patients - Principles of office setup, including design, layout, charting and equipment needs - Principles of hiring and managing support staff personnel (e.g. nurses, assistants, secretaries etc.) b
**Health Advocate**
- Critically evaluate and perform common preventative care interventions and services - Complete the steps required to request coverage for specific drugs not routinely covered by provincial health care plans - Identify and direct patients to appropriate hospital, community and government resources available for patient care (e.g. home care, social work) - Identify barriers to health care resources (e.g. financial, social, physical) for individual patients
**Professional**
- Recognize, prevent and respond to unprofessional behaviour (e.g. intimidation, harassment) by other health care providers - Describe potential threats to medical professionalism posed by conflict of interest (e.g. collaboration with pharmaceutical industry, accepting gifts etc.)
**Scholar**
- Effectively utilize information technology to access medical information and support his/her own education - Pose an appropriate learning question, conduct and document a systemic search for evidence, and integrate the findings into practice - Facilitate the learning of students/residents in the ambulatory setting - Discuss a strategy for lifelong learning, including documenting and recording Continuing Professional Development credits - Apply knowledge of study design and statistical methods when critically appraising clinical studies

b indicates competencies that would have been missed if recent graduates were not included

**Table 4:** Competencies classified as level three priorities

**Medical Expert**
- Demonstrate medical expertise when providing pre-pregnancy counselling and managing medical complications of pregnancy
**Manager**
- Discuss insurance and legal services required by a physician - Balance the allocation of finite health care resources with optimal patient care - State the necessary professional requirements (e.g. licensure, fellowship, membership) to start a practice in his/her province b - Principles of personal and professional financial management (including leasing, accounting, partnerships, incorporation) b - Evaluate practice opportunities with an understanding of job market characteristics, interviewing skills and negotiating principles - Prepare and maintain an effective curriculum vitae
**Health Advocate**
- Appropriately advocate on behalf of a patient to his/her workplace or school b - Identify opportunities for advocacy, health promotion and disease prevention within his/her practice community b
**Professional**
- Recognize, and respond to, other health care professionals in need (e.g. substance abuse problems) - Describe the roles of the Canadian Medical Protective Agency (CMPA) and provincial regulatory bodies (e.g. College of Physicians)
**Scholar**
- Describe the Maintenance of Certification (MOC) program required by the Royal College of Physicians and Surgeons of Canada (RCPSC) for renewal of fellowship

b indicates competencies that would have been missed if recent graduates were not included
